# Supporting early-career women researchers: lessons from a global mentorship programme

**DOI:** 10.1080/16549716.2022.2162228

**Published:** 2023-01-27

**Authors:** Vanessa Brizuela, Joy J. Chebet, Anna Thorson

**Affiliations:** UNDP/UNFPA/UNICEF/WHO/World Bank Special Programme of Research, Development and Research Training in Human Reproduction (HRP), Department of Sexual and Reproductive Health and Research, World Health Organization, Geneva, Switzerland

**Keywords:** Mentorship, research capacity strengthening, low and middle income countries (LMICs), sexual and reproductive health and rights (SRHR), research, women

## Abstract

Mentorship is an important contributor to strengthening research capacity among health researchers. Formal mentorship programmes, targeting women mentees can help mitigate some of the gendered power dynamics and can also help early career researchers learn from others’ experiences of navigating these challenges. In 2020, the UNDP/UNFPA/UNICEF/WHO/World Bank Special Programme of Research, Development and Research Training in Human Reproduction at the World Health Organization launched a mentorship programme geared towards early career women researchers. This paper describes the process of designing and implementing a mentorship programme for early career women sexual and reproductive health and rights researchers from low- and middle-income countries including valuable lessons learned vis-à-vis existing evidence. Some of these findings have been incorporated into iterations of the programme launched in 2022. Critical points include: ensuring considerations for language and geographical distribution; allowing mentees to participate in the matching process; providing training and opportunities to network and learn from other participants; offering the support and structure for developing these relationships. Providing women researchers with the tools – through mentorship – to navigate the unique challenges they face in their career journeys, can have a lasting impact on research capacity. Countries and institutions committed to strengthening research capacity need to focus on the holistic growth and motivation of individuals in a way that ensures gender equality.

## Introduction

Formal mentorship programmes can be beneficial for both mentees and mentors, as long as the relationship is based on positive values [1–3]. Mentorship can also benefit the institution to which mentors and mentees are affiliated [4]. When researchers are provided with mentorship in support of their academic and professional growth, this can contribute to their motivation to continue working with their institutions, reducing attrition and *brain drain*. For mentors, the opportunity to ‘give back’ through supporting early career researchers can contribute to overall work satisfaction [4].

Moreover, mentorship is an important aspect contributing to strengthening research capacity among health researchers [1,5]. Mentorship, as a complement to supervision, can help researchers’ professional development, networking, interpersonal skills, and motivation, as well as foster gender equity and inclusion [6,7]. In order for there to be a critical mass of researchers to carry out relevant and significant research, continuous support and sustainability of their growth is needed [8,9]. Fostering a research environment that is evidence-based, includes the use of critical thinking and innovative approaches as means to tackle sensitive issues, such as those relating to SRHR research (e.g. contraception, abortion, gender-based violence), paramount to making strides in improving population health.

While women^1^^1^In this article, we will refer to women as inclusive of any person who self-identifies as a woman. currently make up the majority of the health workforce and health academia, they are still systematically absent or scant in leadership positions [10,11]. This absence is both a reflection of organisational cultures that do not support women’s career advancement and a consequence of upholding societal gender inequalities whereby women carry the largest burden for work in the household, with subsequently fewer senior career opportunities [12,13]. This is seen especially among recent doctoral graduates who may require additional support at the start of their career; many graduating with a PhD do not continue a career in research [14–16]. Further, women researchers oftentimes experience negative impacts of organisational and structural gender inequalities and these permeate into formal and informal mentoring relationships [17,18]. Formal mentorship programmes, targeting same sex mentor-mentees can help mitigate some of the gendered power dynamics and can also help early career researchers learn from others’ experiences of navigating these challenges [19,20].

Mentorship programmes are varied and attempts at evaluating them have been challenging because of the *ad hoc* nature of many of them [2,21]. However, existing literature shows that professionals require training in mentoring and skills-building for effective mentoring, and programmes require clear objectives and format to ensure success [21–24]. This includes considerations for institutional support to providing mentorship which may be in the form of allocated time and resources to provide mentorship as well as in providing an enabling environment for mentorship [19,25].

In 2020, the UNDP/UNFPA/UNICEF/WHO/World Bank Special Programme of Research, Development and Research Training in Human Reproduction (HRP) at the World Health Organization (WHO) launched a mentorship programme geared towards early career women researchers. For this initiative, mentorship was defined as distinctly different to supervision (see Box 1). The programme was channelled through the HRP Alliance, an initiative entrusted with strengthening SRHR research capacity, particularly in low- and middle-income countries (LMIC) [26]. With a strategy focusing on seven research institutions as regional research capacity strengthening (RCS) hubs across different countries and geographical areas, the HRP Alliance is supporting institutions and researchers across the world in pursuit of improved SRHR [27]. In this paper, we describe the process of designing and implementing a mentorship programme for early career women SRHR researchers from LMICs and the steps taken to monitor and evaluate the programme, as well as valuable lessons learned vis-à-vis existing evidence.

**Table:** **Box 1.** Characteristics of mentors and supervisors*.

Mentors …	Supervisors …
… support professional development	… provides technical guidance on content of work
… foster effective leadership and communication skills	… co-develop and support research, including research outputs
… advocate for equity, diversity, and inclusion	… offer academic career advice based on personal or professional experience
… promote and share networking opportunities	… offer organisational guidance relating to the institutional norms and rules
… offer career advice based on personal skill-building	… manage the research team in pursuit of common goals
… provide different viewpoints, advice, and information based on personal knowledge, experience, and expertise	… hold power over academic/employment advancement or promotion
… offer support in navigating personal life challenges impacting professional growth	… foster visibility and exposure to broader professional network

Note: *not an exhaustive list.

## The HRP Alliance mentorship programme for early career women researchers

Documented successful examples of mentorship programmes included a holistic perspective whereby both topical and research methodology expertise were coupled with personal and professional skills [23–25]. The HRP Alliance mentorship programme focused on the latter, since the former was already being covered through RCS activities led by the hubs. By aiming to match early career women researchers with more experienced researchers, we sought to provide the needed support that many women face at the start of their careers, beyond academic supervision. For the pilot of this programme, we focused solely on women, both for mentors and mentees yet in further iterations, based on suggestions by pilot participants, men were invited to apply for the role of mentors. The initiative included an initial year-long structured programme with recurring targeted training and support to cement the foundation for what we envisioned as long-term mentoring relationships.

### Focus on women

Pervasive gender inequality continues to permeate both health outcomes as well as health research [28,29]. Women’s health issues are understudied and fewer women researchers make it to senior leadership positions in academia [30,31]. This is particularly evident in societies and environments where gender norms pose challenges for women’s career advancement and where sexism is still strong [32]. By providing women with additional support in soft skills needed for progressing in their research careers (from strengthening their communication and networking skills to overcoming daily barriers tied to their research projects), this programme aimed towards bringing women one step closer to gender equality in SRHR research [33].

### The making of the programme

**Figure 1.**
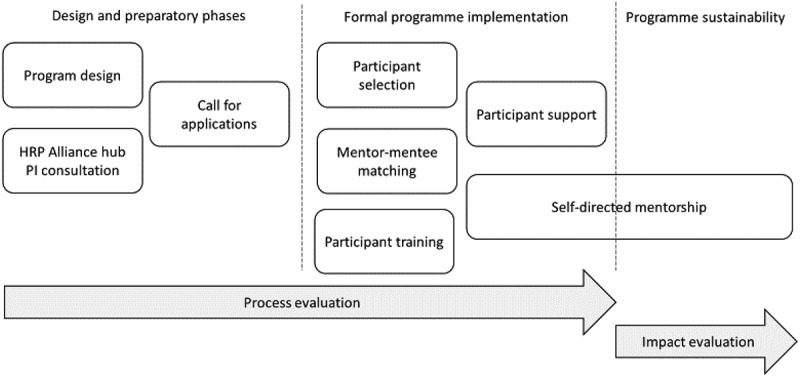
Phases of HRP Alliance mentorship programme.

With input from the principal investigators (PIs) of the seven regional RCS hubs for the HRP Alliance, we launched a pilot programme with a small number of pairs such that individualized support could be provided throughout, as well as to allow them access to the research methods courses offered by the hubs. We contracted a company (AO), experienced in coaching and mentorship, to support the implementation of the year-long programme. The company developed trainings and facilitated workshops for mentors and mentees to discuss and engage with different aspects relating to the mentorship relationship. Figure 1 graphically depicts the different phases of the programme development and design.

### Participants

We issued an open call for applications for the roles of mentors and mentees using HRP’s social media platforms and through directed outreach via email to HRP Alliance partners. An online application form was developed and opened in late 2020. A total of 15 people applied for the role of mentors and 26 for the role of mentee. Mentors had to have proven experience in SRHR research (e.g. as evidenced through publications), express desire and commitment to participate in the year-long formal programme, and participate in the training programme. Mentees had to be from a LMIC, while there were no country restrictions for mentors. Matching was conducted internally by the implementing team (HRP Alliance staff and consultants, HRP Alliance hub PIs, and contractors from AO) based on research interests, professional and personal experiences, and career path aspirations. Two of the mentor applicants were unable to complete the requirements, therefore a total of 13 mentor-mentee pairs took part in the inaugural, pilot programme.

### Implementing the programme

The programme was conceived to be completed primarily online with remote support sessions and mentorship meetings and two in-person activities – an initial training session for mentors and mentees and an end-of-year celebratory event. Because of travel restrictions and health concerns brought on by the COVID-19 pandemic, all activities were conducted online.

During the mentor training session, participants were provided with tools and resources that they could use to help develop rapport with mentees, improve communication, identify bottlenecks for professional development, and frame the relationship between mentors and mentees. Mentees’ training focused on how to optimise their experience – including how to take ownership of their personal development, setting personalized goals, and building trust with their mentors. The support sessions brought participants together, according to role, to share their experiences and collaboratively problem-solve issues that arose in individual mentorship sessions. Table 1 presents the different components and activities and the duration of each. Mentorship sessions between mentors and mentees were left up to each dyad with regard to frequency and duration, as well as content.

**Table 1.** Components of the mentorship programme.

Activity/event	Description	Duration
Launch event	Facilitated discussion for all prospective applicants as mentors and mentees on what is mentoring and what to expect from the programme.	One 90min session
Training session for mentors	Selected mentors were offered tools and resources to support the initiation of their mentorship journey. During the training session, mentors were also offered opportunities for role-playing and practicing using the tools provided.	Full day/two half-days sessions
Training session for mentees	Selected mentees were presented with suggestions and recommendations on how to get the most out of their mentorship relationship.	Half-day session
Support sessions for mentors	Participating mentors brought together to discuss issues arising from mentorship, as well as share challenges and opportunities.	Five 120min sessions
Support sessions for mentees	Participating mentees brought together to network, discuss issues, and problem solve collectively.	Four 120min sessions
Celebration event	Participating mentors and mentees, programme organizers, and other stakeholders participated in an online celebration event to share experiences and successes of programme.	One 120min session
Mentorship meetings*	*Encounters, self-directed by each of the dyads, to discuss topics of their preference using the tools provided through the trainings.*	*Variable*

Note: *Duration, frequency, and content of these meetings was left up to each mentor-mentee pair; these were not overseen by anyone from the implementing team and were left to each pair’s discretion, with a goal to continue past the end date of the formal programme.

### Monitoring and evaluation

Central to understanding whether a pilot programme is effective and impactful is the inclusion of a robust monitoring and evaluation plan. For the pilot programme we included short feedback questionnaires prepared by AO that were sent out to all participants after each of the training and support sessions. In addition, HRP Alliance staff developed online surveys implemented at baseline, mid-way into the programme, and at the end. The goal of these surveys was to get real-time feedback that would allow to course correct and implement changes, as needed. In parallel, a more systematic evaluation including mixed methods (online in-depth interviews and surveys) was put in place, and assessment is ongoing. Similar monitoring activities are being implemented during rollout of the scaled programme.

A mixed methods process evaluation is underway for the pilot programme, through the use of online surveys and semi-structured interviews with participants in the roles of mentor and mentee. The information collected through these mechanisms will be analysed and made public and will reflect on process and outcome measures. See appendix 1 for the logic model used to guide the evaluation. We will continue to engage past (and current) participants over the years to allow for understanding the longer-term impact of the programme on individual career progression and growth.

## Reflexive statement

Implementers of this programme were all women and included three HRP staff (full time staff and consultants, all SRHR researchers) and four AO staff (director and facilitators, all trained in coaching and facilitation). While HRP staff represented high-, middle-, and low-income countries, the entire implementing team was working from a high-income country setting, and in the case of HRP staff, an international organisation funding the HRP Alliance and its activities. We initiated this programme with the belief that women researchers are oftentimes burdened with societal expectations and structures regarding gender, and the anecdotal and experiential knowledge that good mentorship, provided by women, had made a difference in our own research and professional careers. The team at HRP have been involved in SRHR global research individually for decades and the team at AO have been supporting individuals and organisations through mentorship and coaching.

## Discussion and lessons learned

There were many lessons learned from the piloting of this programme. Some relate to development and design issues, while others refer to how the programme was implemented. These were collected through surveys to participants prepared by HRP Alliance, questionnaires after each of the support sessions, and discussions among AO and HRP Alliance secretariat staff that met on a weekly basis to discuss issues, challenges, and opportunities.

### Matching of mentors and mentees

While the original process for matching was based on predefined elements, it was limited by the number of mentor applicants. Most participants to the pilot programme expressed satisfaction with their matched mentor/mentee given alignment with research interests or personal journeys as well as cross-regional pairing. There were, however, requests to allow mentees to take part in the process through having access to some basic information about the potential mentors, in line with others’ experience [34]. Considerations for language fluency were also raised, as the focus on English was an impediment to some participants. Further, both mentors and mentees welcomed a programme aimed at women, especially among mentees who highlighted how the programme provided a space for them to interact with other women researchers and share challenges, struggles, and advice on how to overcome some of the typically gendered obstacles they faced [31,33,35]. However, in further discussions, particularly among mentors, the issue of overburdening women with these activities that are less valued in academia and not considered for career advancement was raised as an issue of concern [36]. Allowing for men to take on mentorship roles would allow for a more, and intentionally balanced vision of mentorship [34], and keeping the role of mentee for women alone would align with our vision to offer the support needed to level the playing field for early career women researchers. Further, while the original plan had been to have this programme focus solely on LMICs for the mentor and mentee roles, given the limited response during the pilot for individuals applying as mentors from LMICs, we broadened the scope to include mentors from HICs while continuing our focus on mentees from research partner institutions for the HRP Alliance.

Based on our experience and feedback provided by participants to the pilot, the scaled mentorship programme launched in early 2022 incorporated several changes:

1. Participants: men were invited to apply for the role of mentor whereas positions for mentees remained dedicated to individuals identifying as women.
2. Language: applications asked for all participants to state the languages preferred for their mentorship relationships.
3. Matching: mentees ranked their first three mentor choices from an anonymised list of mentor profiles. The implementing team then paired dyads based on this preferential ranking, common research interests and language, and aiming for them to be from different regions (as a means to avoid any power differentials between the mentor-mentee pairs and to foster wider networking opportunities).
4. Country/region: mentors from LMICs would be prioritised for the scaled programme to address potential power dynamics that could emerge from the difference in access to resources for mentors in HICs and mentees from LMICs.

### Training and regular support meetings

Training and ongoing meetings were at the core of the HRP Alliance mentorship programme [37]. Providing mentors and mentees with an initial training workshop aimed at making the relationship as successful as possible proved to be beneficial and well received by participants. We proposed a model that provided structure for initiating the relationship while offering room for different forms of relating as a way to foster a lasting mentorship partnership [37,38]. Regular sessions for mentors and mentees separately also provided participants with an opportunity to collectively reflect on issues faced by women researchers, by challenges that SRHR researchers encounter, and to create personal and professional bonds between them. Having tools that mentors and mentees alike could refer to in guiding their meetings and orient their advice proved beneficial to both and seen as one of the unique characteristics of the programme.

While some mentees were able to benefit from methods courses offered by the HRP Alliance hubs, the linkages between mentees and the hubs were not systematic making these opportunities *ad hoc* and reserved to those more closely linked to the hubs. Similarly, mentors expressed being at times lost because they did not have a specific product to work on with mentees (the relationship was, by design, self-directed and distanced from academic supervision) or knowledge of the mentees’ work to better direct them. Our goal was to ensure that the mentorship provided through the programme offered guidance and career advice, tailored to the specific needs of the mentee, and coupled with research methods training offered through the hubs resulting in a holistic support system at the start of their professional journeys [37,39].

Lessons learned from the pilot programme when it came to trainings and regular support sessions were as follows:

1. Baseline training: to accommodate to people’s schedules and the global nature of the programme, the training for mentors was offered twice. An online, self-paced programme was also made available to those who could not attend the live session and applicants that were not selected were also invited to attend, making the resources available to all interested individuals. The initial workshop for mentees was made available to those unable to attend through recordings and copies of materials shared.
2. Professional skill-building: workshops on topics relating to effective communication, networking, solution-based problem solving, among others, were included in the scaled programme, in response to participant demand.
3. Research skills strengthening: to ensure that all mentees are offered specific SRHR research skills and training, they are all invited to take part in any online course being offered by the hubs. This would allow mentees to also become part of the broader network of individuals benefitting from the RCS efforts led by the regional hubs.
4. Ongoing support meetings for mentors and mentees: given the feedback stating that these helped create personal and professional connections, support meetings were offered again ensuring the first one was held early on in the programme.

### Lasting impressions and relationships

The HRP Alliance mentorship programme was devised as a steppingstone in the journey of continued mentorship, the goal being to offer early career researchers with a resource that they could tap on as needed and as their research career in SRHR continued to grow. During the design process, we knew this would likely be influenced by many external factors (timing, chemistry between mentors and mentees, competing interests) [34]. In general, participants mentioned planning to continue the relationship beyond the duration of the structured programme. However, the remote nature and the fact that they had not met in person were seen as barriers to ensure the relationship survived the initial year.

Of note, some of the participating mentors, so positively impacted by the programme, decided to replicate this mentorship model in their own institutions. Having research institutions formalise mentorship programmes shows both a change in organisational culture and in the value of support of professional growth.

In addition to the building of mentor-mentee dyads, the programme was able to build on a broader peer-mentoring network that has the potential to foster future collaboration [35]. Participating mentees helped get the first cohort of mentees organised by creating a mobile messaging group for news and networking, which resulted in a collaboration for joint research funding applications. Similarly, mentors have stayed in touch through mailing groups. Some mentor-mentee pairs decided to collaborate on research projects given their shared interests.

While the long-term, lasting effects of the programme will take time to come to fruition, the ongoing mixed-methods evaluation of the pilot will provide us with a glimpse as to whether the relationships survived a year after the structured programme ended. Of note, many of the mentors that participated in the pilot programme applied to be considered for the scaled programme again, while some of the participating mentees applied to be mentors in the new cohort. These actions speak of the positive impact the programme had on the individuals who participated in the programme and could indicate a desire to ensure that women are being provided with the tools needed to boost the start of their research careers. Since this seemed to be one of the strong points in the pilot programme, no specific changes were introduced in the scaled programme and special attention would be given during the support sessions in the future on the expectation that the relationships outlive the programme.

### Internet connectivity and remote connections

Given the global nature of the programme, compounded by the onset of the pandemic during the design phase, it was devised to be entirely online. This meant relying on good internet connectivity or availability of accessible telephone communications that would allow for meetings between mentors and mentees to occur. Many of the selected mentors were affiliated with educational or academic institutions and had access to reliable online platforms and internet connectivity, yet this was not always the case for mentees who oftentimes used their personal resources for connecting. Great geographical diversity (ranging from Brazil to Viet Nam) meant needing to accommodate to different time zones and calendars.

While the issue of internet connectivity is one of great relevance in today’s changing world [40–42], we have not been able to provide additional resources to support this and evidence has shown that the way in which mentors and mentees communicate is second to the relationship they are able to build [39]. Nevertheless, we took into consideration geographical location and language as two key aspects that were critical to pairing individuals [34,37]. Lessons learned regarding access which will need to be taken into account in the future are:

1. Internet connectivity: application forms and processes should clearly state that a basic requirement for participation in the programme is access to reliable internet. This may mean the individuals need to plan their meetings for times and days that are more feasible for them given their individual possibilities. With more funding, we might be able to provide financial support to participants with limited access to the internet.
2. Geographical span: continue to consider geography, especially with regard to time differences between countries, when selecting and matching participants. Pairs with individuals located in very removed places would have more issues connecting.

### Resources and institutional support

Offering a structured mentorship programme, facilitated by trained experts in the field requires resources, both human and financial. Yet the programme can be implemented at relatively low additional cost to institutions – staff time being the costliest. While neither mentors nor mentees received remuneration for participating, trainings and resources were offered to them free of charge. Relatedly, ensuring that experienced men and women researchers are provided with the time and institutional recognition of their roles as mentors is a critical cornerstone to the success of a mentorship programme [4,36,43–45]. Likewise, if early career researchers are enabled and empowered to build their professional and personal skills beyond the purely technical and methodological expertise they receive through research education, both their positioning as SRHR researchers and their ability to strongly lead in SRHR research will have lasting effects on global SRHR [35,36]. It would seem that the programme might be able to achieve this by continued iterations and through the power of numbers at having more participants claim for the necessary support for mentoring early career researchers.

### Overall considerations on design and implementation

In addition to the lessons identified through this pilot programme, some additional reflections are warranted. First, to acknowledge that this is one model among many; we hope that what we learned can be of use to others trying to develop a mentorship programme for early career women researchers. Second, we understand that as developers of this programme affiliated with WHO and working from high-resourced settings, there are inherent power dynamics underpinning this effort. Third, we recognise that more could have been done to ensure a more equitable participation in the programme. For example, we were unable to attract many mentors from LMICs which may be a reflection of the additional burden and challenges that researchers in LMICs face. These can include issues such as, not being retributed financially or professionally for providing mentorship, or having limited access to high-speed internet connectivity to allow for remote mentorship meetings and participating in the support sessions. Fourth, and relatedly, we were unable to provide support to early career women researchers for conducting research, which we know is one of the barriers that early career researchers face at the start of their professional journeys. And lastly, while this programme offered resources and support to women researchers, breaking entrenched and systematic gender imbalances, shifting organisational culture and societal norms, went beyond the reach of this programme and its objectives. This programme was thought to counteract some of these issues while contributing to change.

## Conclusion

A structured mentorship programme can offer the necessary environment for development of mentors and mentees alike, and for providing the basis for a lasting relationship. Lessons learned from the implementation of a pilot programme aimed at early career SRHR researchers can be useful for others intending to support women with a focus on the holistic growth and motivation of individuals while also combatting gender inequalities.

## Paper context

Women face unique challenges in their career development, which oftentimes hinder or stalling their growth. Mentorship can help overcome of these barriers. Providing researchers with the structured support and practical tools through mentorship at the start of their careers has the potential to have long-lasting impact. A formal global programme can particularly benefit women from low- and middle-income countries in expanding professional networks and benefitting from international collaborations.

## References

[1] Cole DC, Johnson N, Mejia R, McCullough H, Turcotte-Tremblay AM, Barnoya J, et al. Mentoring health researchers globally: diverse experiences, programmes, challenges and responses. Glob Public Health. 2016 Oct 20;11:1093–9. 26234691 10.1080/17441692.2015.1057091 PMC5020346

[2] Libby AM, Hosokawa PW, Fairclough DL, Prochazka AV, Jones PJ, Ginde AA. Grant success for early-career faculty in patient-oriented research: difference-in-differences evaluation of an interdisciplinary mentored research training program. Acad Med. 2016 Dec;91:1666–1675. 27332867 10.1097/ACM.0000000000001263 PMC5177544

[3] Roets L, Van Rensburg EJ, Lubbe J. Faculty’s experience of a formal mentoring programme: the perfect fit. Afr Health Sci. 2019 Aug 21;19:2237. 31656509 10.4314/ahs.v19i2.49 PMC6794521

[4] Charron K, Kalbarczyk A, Martin NA, Combs EA, Ward M, Leontsini E. Building blocks of global health mentorship: motivation, expectations, and institutional support. Ann Glob Health. 2019 Mar 18;85:39. 30896128 10.5334/aogh.1537 PMC6634466

[5] Lansang MA, Dennis R. Building capacity in health research in the developing world. Bull World Health Organ. 2004 Oct;82:764–770. 15643798 PMC2623028

[6] Lescano AG, Cohen CR, Raj T, Rispel L, Garcia PJ, Zunt JR, et al. Strengthening mentoring in low- and middle-income countries to advance global health research: an overview. Am J Trop Med Hyg. 2019 Jan 10;100:3–8. 10.4269/ajtmh.18-0556 PMC6329352 30430982

[7] Sosik JJ, Godshalk VM. Examining gender similarity and mentor’s supervisory status in mentoring relationships. Mentor Tutoring Partnersh Learn. 2005 Apr;13:39–52.

[8] Minja H, Nsanzabana C, Maure C, Hoffmann A, Rumisha S, Ogundahunsi O, et al. Impact of health research capacity strengthening in low- and middle-income countries: the case of WHO/TDR programmes. PLoS Negl Trop Dis. 2011 Oct 11;5:e1351. 22022630 10.1371/journal.pntd.0001351 PMC3191138

[9] Nchinda TC. Research capacity strengthening in the South. Soc Sci Med. 2002 Jun;54:1699–1711. 12113452 10.1016/s0277-9536(01)00338-0

[10] Wennerås C, Wold A. Nepotism and sexism in peer-review. Nature. 1997 May;387:341–343. 9163412 10.1038/387341a0

[11] Jagsi R, Guancial EA, Worobey CC, Henault LE, Chang Y, Starr R, et al. The “gender gap” in authorship of academic medical literature — a 35-year perspective. N Engl J Med. 2006 Jul 20;355:281–287. 16855268 10.1056/NEJMsa053910

[12] Kwamie A, Jalaghonia N. Supporting early-career mentorship for women in health policy and systems research: a vital input to building the field. Health Policy Plan. 2020 Nov 1;35:i4–6. 33165579 10.1093/heapol/czaa105 PMC7649665

[13] Langer A, Meleis A, Knaul FM, Atun R, Aran M, Arreola-Ornelas H, et al. Women and health: the key for sustainable development. Lancet. 2015;386:1165–1210. 26051370 10.1016/S0140-6736(15)60497-4

[14] Peri-Rotem N. Gendered career pathways among Doctoral Graduates in the United Kingdom. Soc Sci. 2019 Nov;8:317.

[15] Anders SV. Why the academic pipeline leaks: fewer men than women perceive barriers to becoming professors. Sex Roles. 2004 Nov 1;51:511–521.

[16] Ahmad S. Family or future in the academy? Rev Educ Res. 2017 Feb 1;87:204–239.

[17] Ramanan RA, Phillips RS, Davis RB, Silen W, Reede JY. Mentoring in Medicine: keys to Satisfaction. Am j med. 2002 Mar;112:112. 10.1016/s0002-9343(02)01032-x 11893387

[18] Sambunjak D, Straus SE, Marušić A. Mentoring in academic medicine: a systematic review. JAMA. 2006 Sep 6;296:1103. 16954490 10.1001/jama.296.9.1103

[19] Hamer DH, Hansoti B, Prabhakaran D, Huffman MD, Nxumalo N, Fox MP, et al. Global health research mentoring competencies for individuals and institutions in low- and middle-income countries. Am J Trop Med Hyg [Internet]. 2018 Nov 14 [cited 2020 Jan 14]. Available from: http://www.ajtmh.org/content/journals/10.4269/ajtmh.18-0558 10.4269/ajtmh.18-0558 PMC6329357 30430976

[20] Downs JA, Mathad JS, Reif LK, McNairy ML, Celum C, Boutin-Foster C, et al. The ripple effect: why promoting female leadership in global health matters. Public Health Action. 2016 Dec 21;6:210–211. 28123954 10.5588/pha.16.0072 PMC5176041

[21] Weber-Main AM, Shanedling J, Kaizer AM, Connett J, Lamere M, El-Fakahany EE. A randomized controlled pilot study of the University of Minnesota mentoring excellence training academy: a hybrid learning approach to research mentor training. J Clin Transl Sci. 2019 Aug;3:152–164. 31660240 10.1017/cts.2019.368 PMC6799418

[22] Sheri K, Too JYJ, Chuah SEL, Toh YP, Mason S, Radha Krishna LK. A scoping review of mentor training programs in medicine between 1990 and 2017. Med Educ Online. 2019 Jan 1;24:1555435. 31671284 10.1080/10872981.2018.1555435 PMC6327936

[23] Sorkness CA, Pfund C, Ofili EO, Okuyemi KS, Vishwanatha JK, Zavala ME, et al. A new approach to mentoring for research careers: the national research mentoring network. BMC Proc. [Internet]Available from. 2017 Dec [cited 2019 Nov 15];11. doi:10.1186/s12919-017-0083-8 PMC5773914 29375663

[24] Pfund C, Byars-Winston A, Branchaw J, Hurtado S, Eagan K. Defining attributes and metrics of effective research mentoring relationships. AIDS Behav. 2016 Sep;20 Suppl 2:238–248. 27062425 10.1007/s10461-016-1384-z PMC4995122

[25] Katz F, Glass RI. Mentorship training is essential to advancing global health research. Am J Trop Med Hyg. 2019 Jan 10;100:1–2. 10.4269/ajtmh.18-0694 PMC6329355 30430975

[26] Adanu R, Mbizvo MT, Baguiya A, Adam V, Ademe BW, Ankomah A, et al. Sexual and reproductive health research and research capacity strengthening in Africa: perspectives from the region. Reprod Health. [Internet]Available from. 2015 Dec [cited 2017 May 25]];12. doi:10.1186/s12978-015-0055-2 PMC4521375 26226944

[27] Adanu R, Bahamondes L, Brizuela V, Gitau E, Kouanda S, Lumbiganon P, et al. Strengthening research capacity through regional partners: the HRP alliance at the World Health Organization. Reprod Health. 2020 Aug 26;17:131. 32847605 10.1186/s12978-020-00965-0 PMC7448306

[28] George AS, Amin A, García-Moreno C, Sen G. Gender equality and health: laying the foundations for change. Lancet. 2019 Jun 15;393:2369–2371. 31155277 10.1016/S0140-6736(19)30987-0

[29] Ostlin P, Sen G, George A. Paying attention to gender and poverty in health research: content and process issues. Bull World Health Organ. 2004 Oct;82:740–745. 15643794 PMC2623023

[30] Pell AN. Fixing the leaky pipeline: women scientists in academia. J Anim Sci. 1996 Nov 1;74:2843–2848. 8923199 10.2527/1996.74112843x

[31] Casad BJ, Franks JE, Garasky CE, Kittleman MM, Roesler AC, Hall DY, et al. Gender inequality in academia: problems and solutions for women faculty in STEM. J Neurosci Res. 2021;99:13–23. 33103281 10.1002/jnr.24631

[32] Oliveira LD, Reichert F, Zandonà E, Soletti RC, Staniscuaski F. The 100,000 most influential scientists rank: the underrepresentation of Brazilian women in academia. An Acad Bras Ciênc. [Internet]Available from. 2021 Sep 20 [cited 2022 Nov 28];93. http://www.scielo.br/j/aabc/a/D5gw9F6w76KrGFPYNGn35nF/?lang=en 10.1590/0001-3765202120201952 34550208

[33] Reese TA, Harris-Tryon TA, Gill JG, Banaszynski LA. Supporting women in academia during and after a global pandemic. Sci Adv. 2021 Feb 24;7:eabg9310. 33627436 10.1126/sciadv.abg9310 PMC7904251

[34] Jackson VA, Palepu A, Szalacha L, Caswell C, Carr PL, Inui T. “Having the right chemistry”: a qualitative study of mentoring in academic medicine. Acad Med J Assoc Am Med Coll. 2003 Mar;78:328–334. 10.1097/00001888-200303000-00020 12634219

[35] DeCastro R, Sambuco D, Ubel PA, Stewart A, Jagsi R. Mentor networks in academic medicine: moving beyond a dyadic conception of mentoring for junior faculty researchers. Acad Med J Assoc Am Med Coll. 2013 Apr;88:488–496. 10.1097/ACM.0b013e318285d302 PMC3610810 23425990

[36] Campos M. Mentoring away the glass ceiling in academia: a cultured critique edited by Brenda L. H. Marina. Interact UCLA J Educ Inf Stud [Internet]. 2016 [cited 2022 Jun 14]. 12(1). Available from: https://escholarship.org/uc/item/15k8533m

[37] Cho CS, Ramanan RA, Feldman MD. Defining the ideal qualities of mentorship: a qualitative analysis of the characteristics of outstanding mentors. Am j med. 2011 May;124:453–458. 21531235 10.1016/j.amjmed.2010.12.007

[38] Javadi D, Hussain S. Enhancing diversity in public health scholarship: the role of publication mentorship. Health Policy Plan. 2020 Nov 1;35:i1–3. 10.1093/heapol/czaa104 PMC7649664 33165578

[39] Pfund C, House SC, Asquith P, Fleming MF, Buhr KA, Burnham EL, et al. Training mentors of clinical and translational research scholars: a randomized controlled trial. Acad Med. 2014 May;89:774. 24667509 10.1097/ACM.0000000000000218 PMC4121731

[40] Litch field I, Shukla D, Greenfield S. Impact of COVID-19 on the digital divide: a rapid review. BMJ Open. 2021 Oct 1;11:e053440. 10.1136/bmjopen-2021-053440 PMC8520586 34642200

[41] Lai J, Widmar NO. Revisiting the digital divide in the COVID-19 Era. Appl Econ Perspect Policy. 2020 Oct 12. DOI:10.1002/aepp.13104. PMC7675734 33230409

[42] Liu J. Bridging digital divide amidst educational change for socially inclusive learning during the COVID-19 pandemic. Sage Open. 2021 Oct 1;11:21582440211060810.

[43] Sopher CJ, Adamson BJS, Andrasik MP, Flood DM, Wakefield SF, Stoff DM, et al. Enhancing diversity in the public health research workforce: the research and mentorship program for future HIV vaccine scientists. Am J Public Health. 2015 Apr;105:823–830. 25122028 10.2105/AJPH.2014.302076 PMC4358212

[44] Tiedeu BA, Para-Mallam OJ, Nyambi D. Driving gender equity in African scientific institutions. Lancet. 2019 Feb 9;393:504–506. 10.1016/S0140-6736(19)30284-3 30739672

[45] Choi AMK, Moon JE, Steinecke A, Prescott JE. Developing a culture of mentorship to strengthen academic medical centers. Acad Med. 2019 May;94:630–633. 31026234 10.1097/ACM.0000000000002498 PMC6493700

